# Mental health status during COVID-19 pandemic in Fars Province, Iran: timely measures

**DOI:** 10.1186/s12889-020-09928-3

**Published:** 2020-12-07

**Authors:** Arash Mani, Ali Reza Estedlal, Mahsa Kamali, Seyede Zahra Ghaemi, Leila Zarei, Nasrin Shokrpour, Seyed Taghi Heydari, Kamran Bagheri Lankarani

**Affiliations:** 1 Research Center for Psychiatry and Behavioral Sciences, Shiraz University of Medical Sciences, Shiraz, Iran; 2 Health Policy Research Center, Institute of Heath, Shiraz University of Medical Sciences, Shiraz, Iran; 3 English Department, Shiraz University of Medical Sciences, Shiraz, Iran

**Keywords:** COVID-19, Mental health, Trust, Iran

## Abstract

**Background:**

The current corona virus pandemic is acting as a stressor or trauma, which not only threats physical health status, but also threats mental health status and well-being of people. Currently, COVID-19 pandemic is a life-threatening unpredictable condition accompanied with a large number of uncertainties. The present study has mainly aimed to assess mental health and the relevant social factors during this pandemic in Fars province.

**Methods:**

This cross-sectional study was performed on 922 participants in Fars province, Iran, using internet-based data collection technique. All the included participants filled out the General Health Questionnaire (GHQ-28). Moreover, demographic variables and some social factors were evaluated by asking some questions. All the participants were ensured of the confidentiality of the collected data, and willingly completed the questionnaire.

**Results:**

Among the participants, there were 629 women (68.2%) and 293 men (31.2%). The mean age of the participants was 36.98 ± 11.08 years old. Four hundred twenty-five subjects (46.1%) obtained GHQ-28 scores above the cut-off point, and accordingly, they were suspected of having poor mental health statuses. Women, in comparison to men (OR = 2.034, 95%:1.62–3.28), and individuals aged < 50 years old, in comparison to those aged > 50 years old (OR: 4.01 95%:2.15–7.50), have poorer mental health statuses. Trusting on media, health authorities, and cooperation with policy makers, as well as having uncertainty on information about Coronavirus pandemic were also shown to be associated with poor mental health condition (*P* < 0.05).

**Conclusion:**

The present study revealed that the number of those people with suspected poor mental health in Fars province significantly increased compared to a previous study using the same questionnaire. Furthermore, the participants who had less trust in media and policymakers were more prone to mental health problems. Therefore, it can be concluded that supporting people in these life-threatening pandemic crises is of great importance, so the policy makers and media must present reliable and valid information to people as soon as possible.

## Background

Since June 10, 2019, COVID-19 outbreak has infected at least 7.5 million people worldwide. Concurrently, according to the WHO, more than 180,000 infected cases and 8500 deaths were reported in Iran so far [1]. According to the report of Shiraz University of Medical Sciences, since June 10, COVID-19 disease infected more than 7000 cases in Fars Province, Iran; however, the deaths resulted from this rapidly-spreading disease exceeded 108 patients. The rapid increase in the new confirmed cases and borderless spread of the disease have raised great concerns on the trajectory future of the current outbreak [2]. In addition, unclear modes of transmission, its long incubation period, and consequently, the pre-symptomatic spread of COVID-19 disease made the new pandemic a stalking invisible threat [3]. The current Coronavirus pandemic acts as a stressor or trauma, which threats not only physical health, but also mental health status and well-being [4, 5]. Evidence obtained from the observations of mental health consequences and measures adopted during previous viral epidemics revealed that the psychosocial aspect of an infectious disease is as important as its treatment aspect [6–12]. Furthermore, psychological factors play critical roles in adherence to public health measures as such individuals usually cope with the threats of infection as well as its consequent losses [8, 13]. Thus, different countries must adopt measures to reduce the transmission rate of COVID-19. Moreover, they should also work more on individuals’ fears to achieve the goal of having a society free from COVID-19 [14–17]. Accordingly, this needs adopting timely measures before the occurrence of prolonged complications such as anxiety disorders, including panic, obsessive-compulsive disorder, stress, and trauma-related disorders [8]. Consequently, this study was performed to further elaborate on the factors affecting social and individual’s mental health.

The findings of a previous mental health survey conducted in 2015 in Fars province indicated that 22.5% of individuals (i.e., 26.9% of women and 18% of men) were suspected of having mild to severe mental symptoms. Evidently, the prevalence of suspected mental symptoms significantly increases with aging. Moreover, the prevalence of the suspected psychiatric disorders was higher in urban areas (24.3%) compared to the prevalence of similar symptoms in rural areas (18.6%). In this survey, somatization symptoms (37.9%) and anxiety (35.8%) were amongst the most reported symptoms. Additionally, 18.7% of the participants had social dysfunctions, and 10.2% of them had depression symptoms. Finally, epidemiological studies conducted over the past 15 years on the mental health status of the general public in this province revealed that the mental health trend exhibited a gradual improvement from 22.9% in 1999 to 22.5% in the subsequent survey in 2015 [18–20].

Considering the published evidences, it can be stated that stress and challenges play major roles in triggering mental symptoms and cause relapse and/or exacerbation of some major mental disorders such as depression and anxiety [21–28]. Moreover, the unremitting threats and long-term effects of stressful conditions could be even more devastating [29]. From a psychological point of view, the current pandemic can be considered as an exception compared to some other stressors and traumas like war or natural disasters like earthquake [3]. The COVID-19 pandemic is a life-threatening unpredictable condition accompanied with a large number of uncertainties [21, 30]. Furthermore, with the capacity of a sanctioned country to respond to this pandemic under a tough economic condition, the damaging effects of this unique stressor on public and individual’s mental health could be even higher than what is expected, thereby a re-evaluation is required [31]. Accordingly, the main objective of the present study was to assess the mental health status and the associated social factors during the current pandemic in Fars province, southern Iran.

## Methods

Fars Province, with an area of 122,842 km^2^, is located in the south of Iran. The capital city of this province is Shiraz. Notably, Fars is known for its rich Persian cultural and historical backgrounds. Its population is about 4,851,274 people, of whom 3,401,675 live in urban areas (70.1%) and 1,432,355 individuals live in rural areas (29.9%). In total, 50.7% of the province’s population are men, and 49.3% are women [32]. This cross-sectional study was conducted in Fars province during March 26–28, 2020 using the internet-based data collection technique. The population of the present study were urban and rural residents of this province. The participants were randomly selected from the province who had access to the internet. Thereafter, a questionnaire link was sent to them, and they completed the questionnaires. In other words, the researchers had no face-to-face interaction with the included participants. Based on this method, we have sent the questionnaire link to random Fars province’s phone numbers found in the national phone directory, so the questionnaire link was randomly spread.

Although about 2000 individuals viewed and clicked the questionnaire link, only 922 individuals finally completed the task and filled out the general health questionnaire (GHQ-28). This scale is a valid and reliable questionnaire developed by Goldberg and Hillier (1979) [33–35], which was translated to Persian and validated, and then proposed cut off points (individuals with GHQ-28 score more than 23 had poor mental health status) in Iran by Noorbala et al. [36]. GHQ-28 is regarded as a highly accepted survey tool to measure general mental health status, which is widely used in public health studies [37–39]. However, it is inevitable that each screening tool has its own false positive as well as its specificity and sensitivity, especially in online surveys, which are not definite. This questionnaire consists of 28 questions scored using a Likert-scale. Additionally, it contains four subscales and 7 items for each subscale to evaluate somatization, anxiety, social dysfunction, and depression symptoms. In this study, the Cronbach’s alpha for the GHQ-28 was 0.831.

Moreover, the participants completed a demographic questionnaire addressing gender, age, level of education, marital status, and economic status (low, middle, and high incomes regarding the individuals’ self-reported monthly costs). There also were self-reports on the history of cigarette smoking, waterpipe smoking, alcohol abuse, sedative abuse, and any other kind of drug abuse. These variables that were well studied in published evidence, are regarded as major determinants of mental health status [18, 40]. In addition, there were questions about some social factors such as the most common information-gathering media (e.g., national television and radio, satellite, virtual social networks, and the web), trusting on media, health authorities, and cooperation among policy-makers, as well as uncertainty about the reported Coronavirus information. The self-report questionnaire is presented in Table 1. These items were adapted and then modified based on the literature [41–45].

**Table 1:** Questionnaire of self-reported questions

1. What’s your marital status?	1. Single 2.Married 3.widowed/divorced
2. What’s your educational degree?	1. Under-diploma 2.diploma 3. Associate degree 3. Bachelor 4. Master degree or higher
3. Do you have trust in national health authorities on controlling the COVID pandemic?	1. Yes 2. no
2. Do you have trust in cooperativeness of national health authorities?	1. Yes 2. No
3. What’s your information gathering channels for COVID-19?	1. National TV/radio 2. Satellite 3. Social media 4. internet
4. Do you have trust in media information for COVID-19?	1. Yes 2. No
5. Are you certain about the information of COVID-19 in media?	1. Yes 2. No
6. What is the status of your monthly earnings in comparison to your monthly costs?	1. Higher income 2. sufficient 3. lower income
4. Do you smoke cigarette?	1. No, Never 2. Yes, occasional 3. Yes, Persistent
5. Do you smoke waterpipe?	1. No, Never 2. Yes, occasional 3. Yes, Persistent
6. How often do you drink alcoholic beverages?	1. Never 2. Once per month 3. Once per week 4. Multiple times per week 5. everyday
7. How often do you use sedatives?	1. No, Never 2. Yes, occasional 3. Yes, Persistent
8. Do you abuse any illegal drugs (opium, heroin, cannabis, LSD, Amphetamine)?	1. No 2. Yes

### Ethics approval

The research protocol was evaluated and approved by the Ethics Committee at Shiraz University of Medical Sciences (Ethics code: IR.sums.rec.1399.077). All the participants were asked to submit their written consent form to participate in this study before completing the online questionnaires. Furthermore, all of them were ensured of the confidentiality of the collected data, and then completed the questionnaires willingly.

### Statistical analysis

Statistical Package for the Social Sciences Version 19.0 (SPSS Inc., Chicago, IL, USA) was used to analyze the obtained data. Frequency (%) and mean ± standard deviation were also used as descriptive statistics. Chi-square test was also employed to determine the relationship among demographic variables and social factors of Coronavirus pandemic and mental health status. Thereafter, Multiple logistic regression was performed to compute odds ratio (OR) and the corresponding confidence interval (95% CI) for demographic variables and social factors of Coronavirus pandemic. *P* < 0.05 was considered as the statistically significant level.

## Results

Among the participants, there were 629 (68.2%) women and 293 (31.2%) men. The participants’ mean age was 36.98 ± 11.08 years old (ranged from 18 to 77 years old), with 567 participants (61.5%) with the age ranged from 30 to 49 years old.

In this study, 425 subjects (46.1%) received GHQ-28 scores above the cut-off point, and accordingly, they were suspected of having poor mental health. Notably, the distribution of the participants above the cut-off point by gender was significant (36.9% men and 50.4% women, *P* < 0.001). Moreover, considering the cut-off point suggested in the GHQ-28 subscales, the prevalence rates of somatization symptoms, anxiety symptoms, social dysfunctions, and depression symptoms amongst the suspected participants were 6.9, 18.9, 21.8, and 5.6%, respectively.

Furthermore, 54.3% of the participants aged < 30 years old, 48.7% of the participants aged between 30 and 49 years old, and 24.8% of them aged > 50 years old had poor mental health statuses (*P* < 0.001). Table 2 shows the relationship between demographic factors and the status of mental health. Accordingly, age, gender, cigarette, water pipe, and sedatives abuse were indicated to be significantly associated with poor mental health (*P* < 0.05); however, marital status, level of education, economic status (based on self-reports), and alcohol and drug abuses had no correlation with mental health. Trusting on the media, health authorities, and cooperation among policy-makers, as well as uncertainty of information about Coronavirus pandemic were also correlated with poor mental health (Table 2).

**Table 2:** The relationship of demographic features, drug abuse variables and information about corona virus with mental health status

	Mental disorders		*P* value
	No	Yes	
Age			
Less than 30	95 (45.7)	113 (54.3)	< 0.001
30–49	291 (51.3)	276 (48.7)	
50 and more	82 (75.2)	27 (24.8)	
Sex			
Male	185 (63.1)	108 (36.9)	< 0.001
Female	312 (49.6)	317 (50.4)	
Marital Status			
Single	141 (50.4)	139 (49.6)	0.181
Married	347 (55.9)	274 (44.1)	
Divorced or Widowed	9 (42.9)	12 (57.1)	
Education			
Under diploma	38 (53.50)	33 (46.5)	0.111
Diploma	100 (58.1)	72 (41.9)	
Associate Degree	37 (56.9)	28 (43.1)	
Bachelor	176 (57.1)	132 (42.9)	
Master degree or higher	146 (47.7)	160 (52.3)	
Economic			
Lower income	90 (50.3)	89 (49.7)	0.458
Sufficient	249 (55.7)	198 (44.3)	
High income	158 (53.4)	138 (46.6)	
Cigarette			
No	433 (55.4)	348 (44.6)	0.037
Occasional	39 (41.5)	55 (58.5)	
Continual	25 (54.3)	21 (45.7)	
Water pipe			
No	438 (55.4)	353 (44.6)	0.047
Occasional	106 (88.3)	14 (11.7)	
Continual	3 (30)	7 (70)	
Sedative			
No	422 (58.3)	302 (41.7)	< 0.001
Occasional	63 (38.7)	100 (61.3)	
Continual	11 (34.4)	21 (65.6)	
Drug abuse			
No	481 (54.4)	403 (45.6)	0.129
Yes	14 (41.2)	20 (58.8)	
Alcohol			
No	397 (55)	325 (45)	0.211
Yes	100 (50)	100 (50)	
The most information gathering channels for COVID-19			
Television or radio national			
No	215 (50.7)	209 (49.3)	0.072
Yes	282 (56.6)	216 (43.4)	
Satellite			
No	137 (55.9)	108 (44.1)	0.461
Yes	360 (53.2)	317 (46.8)	
Virtual social networks			
No	350 (54.6)	291 (45.4)	0.521
Yes	147 (52.3)	134 (47.7)	
Web			
No	455 (54.2)	384 (45.8)	0.527
Yes	42 (50.6)	41 (49.4)	
Trust to the media			
No	138 (43.8)	177 (56.2)	< 0.001
Yes	359 (59.1)	248 (40.9)	
Trust to health authority			
No	244 (47.3)	272 (52.7)	< 0.001
Yes	253 (62.3)	153 (37.7)	
Trust of cooperation between policy makers			
No	304 (48.3)	326 (51.7)	< 0.001
Yes	193 (66.1)	99 (33.9)	
Uncertainty to information about corona			
No	196 (60.5)	128 (39.5)	0.003
Yes	301 (50.3)	297 (49.7)	

Logistic regression analysis revealed that the female participants had poorer mental health (OR: 2.034; 95% CI: (1.62–3.28) compared to the male ones. Moreover, the young participants had worse mental health statuses (OR = 4.01: 95% CI: 2.15–7.50), compared to those aged > 50 years old. Besides, the participants who occasionally smoked cigarette (OR = 19.1; 95% CI: 1.12–3.24) or used sedatives (OR = 2.46; 1.65–3.66) had higher GHQ scores compared to those with no history of drug abuse. The participants who had no trust in media (OR = 1.68; 95% CI: 1.21–2.35) or in cooperation among policy-makers (OR = 1.56; 95% CI: 1.04–2.35), and those with uncertainty about the reported coronavirus information (OR = 1.37; 95% CI: 1.00–1.88) also had higher GHQ scores (Table 3).

**Table 3:** Association between mental health status with demographic variables, drug abuse and information about corona virus base on univariate and multiple logistic regression

	Unadjusted Odds ratio	95% CI for Unadjusted odds ratio	***P*** value	Adjusted Odds ratio	95% CI for adjusted odds ratio	*P* value
Age						
Less than 30	3.61	(2.16–6.04)	< 0.001	4.01	(2.15–7.50)	< 0.001
30–49	2.88	(1.81–4.59)	< 0.001	2.87	(1.71–4.82)	< 0.001
50 and more	1	–	–	1	–	–
Sex						
Male	1	–	–	1	–	–
Female	1.74	(1.31–2.31)	< 0.001	2.30	(1.62–3.28)	< 0.001
Marital Status						
Single	1	–	–	1	–	–
Married	0.801	(0.60–1.06)	0.124	1.26	(0.87–1.86)	0.223
Divorced or Widowed	1.353	(0.55–3.31)	0.509	1.42	(0.49–4.10)	0.517
Education						
Under diploma	1	–	–	1	–	–
Diploma	0.83	(0.48–1.45)	0.509	0.97	(0.52–1.82)	0.923
Associate Degree	0.87	(0.44–1.72)	0.690	1.00	(0.47–2.13)	0.998
Bachelor	0.86	(0.51–1.45)	0.579	1.00	(0.56–1.82)	0.987
Master degree or higher	1.26	(0.75–2.12)	0.378	1.30	(0.71–2.37)	0.397
Economic						
Lower income	1	–	–	1	–	–
Sufficient	0.80	(0.57–1.14)	0.219	0.88	0.59–1.30)	0.510
High income	0.88	(0.61–1.28)	0.512	1.14	(0.75–1.75)	0.533
Cigarette						
No	1	–	–	1	–	–
Occasional	1.76	(1.14–2.71)	0.011	1.91	(1.12–3.24)	0.017
Continual	1.05	(0.58–1.90)	0.885	1.19	(0.56–2.54)	0.647
Water pipe						
No	1	–	–	1	–	–
Occasional	1.466	(0.99–2.16)	0.052	1.46	(0.92–2.31)	0.109
Continual	2.895	(0.74–11.28)	0.125	2.07	(0.48–8.93)	0.327
Sedative						
No	1	–	–	1	–	–
Occasional	2.22	(1.57–3.140	< 0.001	2.46	(1.65–3.66)	< 0.001
Continual	2.67	(1.27–5.62)	0.010	2.52	(1.10–5.77)	0.029
Drug abuse						
No	1	–	–	1	–	–
Yes	1.17	(0.85–3.42)	0.133	1.27	(0.562–2.88)	.564
Alcohol						
No	1	–	–	1	–	–
Yes	1.22	(0.89–1.67)	0.211	0.88	(0.58–1.33)	0.543
The most information gathering channels for COVID-19						
Television or radio national						
No	1	–	–	1	–	–
Yes	1.27	(0.98–1.65)	0.073	1.12	(0.77–1.63)	0.556
Satellite						
No	1	–	–	1	–	–
Yes	1.12	(0.83–1.50)	0.461	1.05	(0.71–1.54)	0.811
Virtual social networks						
No	1	–	–	1	–	–
Yes	1.03	(0.94–1.13)	0.521	1.03	(0.69–1.52)	0.901
Web						
No	1	–	–	1	–	–
Yes	0.87	(0.55–1.36)	0.527	1.35	(0.78–2.345)	0.291
Trust to the media						
No	1.86	(1.41–2.45)	< 0.001	1.68	(1.21–2.35)	0.002
Yes	1	–	–	1	–	–
Trust to health authority						
No	1.84	(1.41–2.40)	< 0.001	1.22	(0.85–1.76)	0.283
Yes	1	–	–	1	–	–
Trust of cooperation between policy makers						
No	2.09	(1.57–2.79)	< 0.001	1.56	(1.04–2.35)	0.032
Yes	1	–	–	1	–	–
Uncertainty to information about corona						
No	1.51	(1.15–1.99)	< 0.001	1	–	–
Yes	1	–	–	1.37	(1.00–1.88)	.053

The relationships of marital status, level of education, economic status, waterpipe smoking, drug and alcohol abuses, and trust in health authorities with mental health status were not statistically significant (*P* > 0.05).

## Discussion

The findings of this study revealed that almost half (46.1%) of the included participants were suspected to have poor mental health statuses in Fars Province. Compared to the general mental health surveys performed in Fars province, the prevalence rate of mental symptoms was almost doubled during coping with the COVID-19 pandemic (with the prevalence rate of 22.5% in 2015 to 46.1% in this study) [18, 19]. In this study, it was shown that women have higher rates of mental health problems (50.4%) compared to men (36.9%) with OR of 2.034. A review conducted on the past studies in Fars province indicated that the prevalence rate of mental symptoms was higher among female subjects with lower OR (1.515 in 2015), compared to the present study [19, 46]. The findings of this study have revealed that older participants had better mental health statuses, and the highest prevalence of mental symptoms also belonged to the age group ≤29 years old (54.3%). This finding is inconsistent with the findings of previous mental health surveys conducted under normal conditions [18, 19, 46, 47]. In the most recent general heath survey in 2015 in Fars, Noorbala et al. have reported that individuals at the age group of ≥65 years old were the most susceptible ones to mental health problems (33.7%). Additionally, the results show poorer mental health statuses among cigarette, water pipe, and psychiatric drug’s users, compared to the other patients’ groups. This finding is in line with those of most studies in Iran [48–51]. The inconsistencies in statistics might be due to social, economic, and political structures of the country and the disease-related factors. Therefore, it is important to consider this deterioration in mental health status.

Primarily, concerning the social culture, greeting, and etiquette of Iranians, the pandemic and the related containment measures such as commuting restrictions, social distancing, missing face-to-face connections, and practicing self-isolation undeniably have detrimental impacts on their psychosocial health statuses. In addition, these factors act as potential risk factors for several mental disorders, including schizophrenia, generalized anxiety disorder, and major depression [3, 22, 23, 29, 30, 52]. Furthermore, the coincidence of public sanctions and the Persian New Year’s (i.e., Nowruz) two- week holidays made some negative mental effects of such restrictions even more devastating. Additionally, the closure of holy shrines; worship places; and cultural and educational sites such museums, schools, cinemas, and theatres eliminated the benefits of social support, as they directly contribute to public health and national identity [53].

From an economical perspective, Iran’s capacity to respond to the virus has been remarkably delayed by economic sanctions [54]. With the greatest sanctions ever imposed on Iran, the country’s health system cannot adopt the same measures as the other countries to strengthen responses and also to provide essential medical equipment and medications. Consequently, the coincidence of COVID-19 pandemic and this economic crisis in Iran has not only made funding adequate prevention, diagnosis, and treatment of COVID-19 problematic, but it has also affected about six million patients with complex and chronic illnesses [31]. These restrictions and lack of support ruined the public trust on governmental policies, measures, and information as such high levels of stress and concerns aroused. Altogether, this scenario clearly explains the findings of the present study. In this case, individuals feel miserable, so they more tend to abuse cigarettes and sedatives, rather than trying to cope with the problem.

Reviewing past studies on major health crises highlights the significance of trust between the public and public health authorities [41, 42]. During these kinds of disasters, the rapid growth of contradictory sources of information, inability of health authorities to provide reliable health-related resources, and the uncertainty of the situation arise skepticism to the health authorities and their cooperativeness, which subsequently result in a critical decrease in the public’s trust on health authorities [41]. In the present study, we supposed that this process might be associated with a poor mental health status.

In addition to the social disturbances and economic pitfalls, the data obtained from previous SARS, MERS, and Ebola outbreaks verified the findings of this study. The facts about the disease, including the official confirmation of human- to-human transmission, its severity and mortality rate, and lack of effective treatments or vaccines to prevent that may also generate uncertainty, anxiety, and a feeling of stigmatization, which consequently evoke some health-risk behaviors such as the enhanced smoking or drinking, drug misuse, recklessness, obsessive-compulsive symptoms, aggression, and suicide commitment [8, 55]. Finally, these behaviors may potentially decrease adherence to the treatment.

Moreover, the public fear and anxiety during the first weeks of the crisis provoked impulsive reactions in people, and spread the feelings of fear, anger, and complaints. Thereafter, they sought for relevant information from various sources and contributed to the circulation of a plethora of misinformation via social media. Thereafter, the information overload, which is referred to as “Misinfodemics”, created uncertainties, concerns, and high levels of anxiety [21, 56]. The unpredictable future of this wasteful news’ consumption along with the risk of fake news ruining everything more quickly than the virus itself would distort the perception of the risk and impair the trust on any kind of news, which result in misunderstanding of health messages [15, 56].

Last but not least, the lack of proper online welfare facilities, concerns about more vulnerable groups of people, and their unique emotional responses intensified the urge for taking decent measures. Firstly, frontline health professionals, who have close contacts with the infected patients, are imposed by excessive workload, isolation, and discrimination; thus, they are highly vulnerable to experience higher degrees of psychological stress, physical exhaustion, fear, emotion disturbance, and some sleep problems [23]. Secondly, the cases with the confirmed and suspected COVID-19 disease may experience fear of severe complications and contagion. Consequently, they may experience loneliness, denial, anxiety, depression, insomnia, and despair, which may in turn reduce their adherence to the treatments. In addition, some of these cases may even experience the increased risks of aggression and committing suicide [9, 57]. finally, high-risk individuals face difficulties in receiving appropriate treatments because of isolation measures, which may thus end up with disease relapse, irreversible complications, and even a higher susceptibility to COVID-19 along with higher rates of morbidity and mortality [8, 58, 59].

### Recommendations

1. Note that social interaction is of paramount significance. If the movements are restricted, it would be preferable to keep regular contacts with individuals via telephone and online channels. Also, using social media accounts to promote positive and hopeful stories is another proposed technique.
2. Keep an eye on mental health statuses in younger participants. According to the findings, the young individuals’ mental health statuses should remain as a priority. Besides, education disruption can impose an additional pressure on the young people. School routines provide mental health support for the young. Parental awareness, new daily routine, and regular communication with friends and family, as well as online education are the main measures recommended for this group of population.
3. Establish online health services. The provision of online platforms for diagnosis and psychological counselling for health workers, patients, and new cases is indispensable. Online psychological self-help intervention systems, including online cognitive behavioral therapy, can also be helpful in the treatment of depression, insomnia, etc. Moreover, developing regular online articles, videos, animations, and audios would be of great values in improving the quality and effectiveness of mental health interventions. Finally, running multiple and regular health surveys on general and special population groups are of a significant importance to enable health policymakers allocating health resources and yielding timely measures.
4. Stop the misinfodemics. It is essential for individuals to minimize newsfeeds. Monitor the time spent on news that make them feel anxious or distressed. It is recommended to seek the latest information via reliable media at specific times of the day. Health authorities should provide people with reliable and valid information via reliable media as soon as possible. Moreover, the modification of misinformation in any media would help both people and news providers to provide more reliable contents.

### Limitations of the study

This study had several limitations. Firstly, because of this disease’s outbreak, we failed to conduct face-to-face interviews. Online data collection has many advantages and limitations. The absence of an interviewer, inability to reach challenging groups of people such as respondents with no access to the internet or elderly people, and lower response rates are some of the notable limitations. In our internet survey, a majority of the respondents were women and young people. This may be due to the fact that, this group of population has better access to the internet, have more free time to complete the questionnaires, or they are more concerned with their mental health statuses. Secondly, the findings were limited due to the use of convenience sampling, which could not consequently reflect the overall condition of Iran’s population. Finally, regarding the fact that this study was cross sectional, we have found some associations among various variables and then tried to consider several confounder variables. However, it is evident that there are some other variables that affect both the dependent variables and independent variables, which consequently cause a spurious association. This is one of the limitations of this study and we proposed it to be considered in future studies.

Besides what was proposed earlier, some variables might also have effects on mental health, which we cannot consider them in our research. Accordingly, the main one was the total impact of the US sanctions, which was discussed before in a recent study [31], so the Iranian population became too weak in coping with the current COVID-19 outbreak. Another point that should be considered in future studies is that we did not evaluated any kind of health problems (somatic or mental) unrelated to COVID-19 outbreak.

## Conclusion

Compared to the general mental health surveys in Fars province, the number of individuals with suspected poor mental health statuses in Fars province has almost been doubled during coping with COVID-19 pandemic. According to this survey, during this pandemic, the young people (< 50 years old) and women are more prone to mental problems, so they require special attention. In addition, the participants who have less trust on the media and policy-makers were shown to be more prone to mental problems. Thus, it can be concluded that, in these life-threatening pandemic situations, cooperation of policy-makers and the reliability and validity of the news in media play critical roles in individuals’ mental health statuses.

## Data Availability

The datasets used and/or analyzed during the current study available from the corresponding author on reasonable request.

## Abbreviations

OR: Odds ratio
GHQ: General health questionnaire
